# Glomerular and Tubular Functions in Transfusion-Dependent Thalassemia

**DOI:** 10.4274/tjh.2018.0083

**Published:** 2018-05-25

**Authors:** Pathum Sookaromdee, Viroj Wiwanitkit

**Affiliations:** 1 TWS Primary Care Center, Bangkok, Thailand; 2 Hainan Medical University, Department of Tropical Medicine, Haikou, Hainan, China

**Keywords:** Glomerular, Tubular, Thalassemia

## To the Editor,

Annayev et al. [1] reported their interesting observations in the publication entitled “Glomerular and Tubular Functions in Children and Adults with Transfusion-Dependent Thalassemia” (TDT). They concluded that “subclinical renal injury may be present in TDT patients” [1]. We would like to share ideas and experiences from our setting in Southeast Asia where transfusion-dependent beta-thalassemia is very common. Renal dysfunction is not uncommon in our thalassemic patients and the degree of dysfunction varies [2]. In fact, the varying degree of renal dysfunction in thalassemia patients is well known [3,4]. Patients with different variants of thalassemia have different degrees of renal dysfunction [3,4,5]. Ong-ajyooth et al. [5] noted that “The mechanism leading to the damage is not known but it might be related to increased oxidative stress secondary to tissue deposition of iron, as indicated by the raised levels of serum and urine MDA”. Improved renal function is also observed after stem cell transplantation therapy [6].
