# Supplementary material for: Glomerular and Tubular Functions in Transfusion-Dependent Thalassemia
Source: Turk J Haematol. 2018 May 25;35(2):137–8. doi: 10.4274/tjh.2018.0083 (PMC5972338; doi:10.4274/tjh.2018.0083)
Supplement: Related texts

# Reply to the Authors:

**Authors:** Zeynep Karakaş, Serap Karaman

## To the Editor,

We thank Drs. Sookaromdee and Wiwanitkit for their interest and contribution to our article. There is a growing evidence of renal dysfunction in patients with thalassemia. Although the process is multifactorial (the disease itself with regular transfusion, iron accumulation in the parenchyma and toxicity of chelators), oxidative stress seems to be the main mechanism of renal damage. Several studies have shown the beneficial effects of antioxidants (curcumin, glutamine) in prevention of chemotherapy-induced nephrotoxicity by decreasing oxidative damage. Considering the significantly increased life expectancy of thalassemia patients with long-term complications, we think the role and effects of antioxidant treatments in routine follow-up of the thalassemia patients should be investigated in prospective studies.
